# Environmental Polychlorinated Biphenyl Exposure and Breast Cancer Risk: A Meta-Analysis of Observational Studies

**DOI:** 10.1371/journal.pone.0142513

**Published:** 2015-11-10

**Authors:** Jingwen Zhang, Yue Huang, Xiaoling Wang, Kun Lin, Kusheng Wu

**Affiliations:** Department of Preventive Medicine, Shantou University Medical College, Shantou, Guangdong, China; The University of Iowa, UNITED STATES

## Abstract

**Background:**

Association between polychlorinated biphenyl (PCB) exposure and breast cancer risk has been widely studied, but the results remain controversial. We performed a meta-analysis to evaluate the evidences from observational studies on PCB exposure and breast cancer risk.

**Methods:**

Relevant studies with data on internal PCB dose were identified from PubMed, EMBASE, CBM and CNKI databases through November 2014. Multivariable-adjusted odds ratio (OR) with 95% confidence intervals (CIs) were applied to assess the association between PCB exposure and breast cancer risk. Heterogeneity test, sensitivity analysis, subgroup analysis and publication bias test were also performed. To further explore the association between specific groups of PCB congeners and breast cancer, we examined the PCB congeners classified, according to their structural, biological and pharmacokinetics properties, as group I (potentially estrogenic), group II (potentially anti-estrogenic and immunotoxic, dioxin-like), and group III (phenobarbital, CYP1A and CYP2B inducers, biologically persistent).

**Results:**

Of 660 studies screened, 25 studies which met criteria were selected, involving a total of 12866 participants (6088 cases and 6778 controls) from eight countries. The results showed that the risk of breast cancer was associated with group II (OR = 1.23, 95% CI: 1.08–1.40) and group III (OR = 1.25, 95% CI: 1.09–1.43) PCBs, but not with group I (OR = 1.10, 95%CI: 0.97–1.24) PCBs or total PCB exposure (OR = 1.09, 95%CI: 0.97–1.22).

**Conclusions:**

Our meta-analysis based on the selected studies found group II and group III PCB exposure might contribute to the risk of breast cancer. More studies in developing countries with higher PCB levels are needed, as well as studies to explore the relationships between mixtures of organochlorine compounds and breast cancer risk.

## Introduction

Breast cancer is the most common cancer among women in the world, there were nearly 1.67 million new cancer cases diagnosed (25% of all cancers) in 2012 and a slight majority of cases occurred in women in developing countries [1]. During the recent past, the rapid rising incidence of breast cancer in developing countries suggested that ongoing environmental change might be a primary contributor [2]. The association between breast cancer risk and industrial development, historically and worldwide, is one indicator of modifiable risk [3]. Risk factors for breast cancer can be classified into four broad categories: genetic/familial, reproductive/hormonal, lifestyle, and environmental. However, these known risk factors explain only a small fraction of cases. More and more studies indicate that the effects of hormones and environmental factors are becoming increasingly important as breast cancer risk factors [4–7].

During the past four decades, attention has been on the potential factor of environmental pollutants such as polychlorinated biphenyls (PCBs), which were classified by the International Agency for Research on Cancer (IARC) in 2013 as carcinogenic to humans (Group 1) on the basis of sufficient evidence of carcinogenicity in humans and experimental animals [8]. PCBs are a class of aromatic compounds comprising 209 congeners, which were widely used as dielectric fluid in capacitors and transformers, and to a lesser extent in building materials. Although PCB production was banned in most countries by the 1980s, PCBs have become ubiquitous environmental pollutants, because of persistence and bioaccumulation [9, 10]. They are still found in atmosphere, soil, rivers, lakes, fish, wildlife, animals, and humans. Humans are exposed to PCBs mainly from ingesting PCB-contaminated food (particularly sportfish and wildlife) or from breathing PCB contaminated air [11, 12]. Recently, renewed production of PCBs has been reported in the Democratic People’s Republic of Korea [13].

Mass poisonings occurred in Japan in 1968 [14] and in Taiwan in 1979 [15], each involving about two thousand people who had consumed PCB-contaminated cooking oil and then developed symptoms such as fatigue, chloracne, pigmentation of nails, skin, and gums, and hypersecretion of eyelid oil glands. The clinical syndrome was later called Yusho or Yucheng, “oil disease” in Japanese and Chinese, respectively. PCBs potentially target the endocrine system, monohydroxylated PCB metabolites can act as oestrogen agonists or antagonists [16, 8]. Worldwide monitoring programmes have shown that PCBs are present in most samples of human milk [17]. These disruptions may have reproductive, toxic, and carcinogenic consequences [18–21]. In 2007, scientists from the Silent Spring Institute reviewed the previous five years of epidemiologic studies of environmental pollutants and breast cancer, and concluded that epidemiologic studies had strengthened the human evidence that PCB exposure plays a role in breast cancer risk [22]. However, the results from epidemiologic studies have been inconsistent. Thus, we conducted a meta-analysis to evaluate the evidence from epidemiologic studies published to date on PCB exposure and the risk of breast cancer by summarizing it quantitatively.

## Materials and Methods

This study was designed, conducted and reported in adherence to the standards of Meta-analysis of Observational Studies in Epidemiology checklist [23]. Two authors (Zhang and Wu) participated in the literature search, study selection, and data extraction independently, and any disagreement was resolved by consensus.

### Search Strategy

PubMed (http://www.ncbi.nlm.nih.gov.pubmed), EMBASE (https://www.elsevier.com/ solutions/embase—biomedical research), CBM (http://libcx.med.stu.edu.cn/) and CNKI (http://epub.cnki.net/kns/default.htm) were searched for studies published through November 2014. The following keywords were used in searching: “polychlorinated biphenyls or PCBs” and “breast cancer or mammary cancer”. Detailed search terms are provided in the supplementary materials (S1 Table). The languages were limited to English and Chinese; no other restrictions were imposed. We also manually searched the reference lists of included articles for original reviews, and searched those for additional relevant studies.

### Selection Criteria

**Fig 1:**
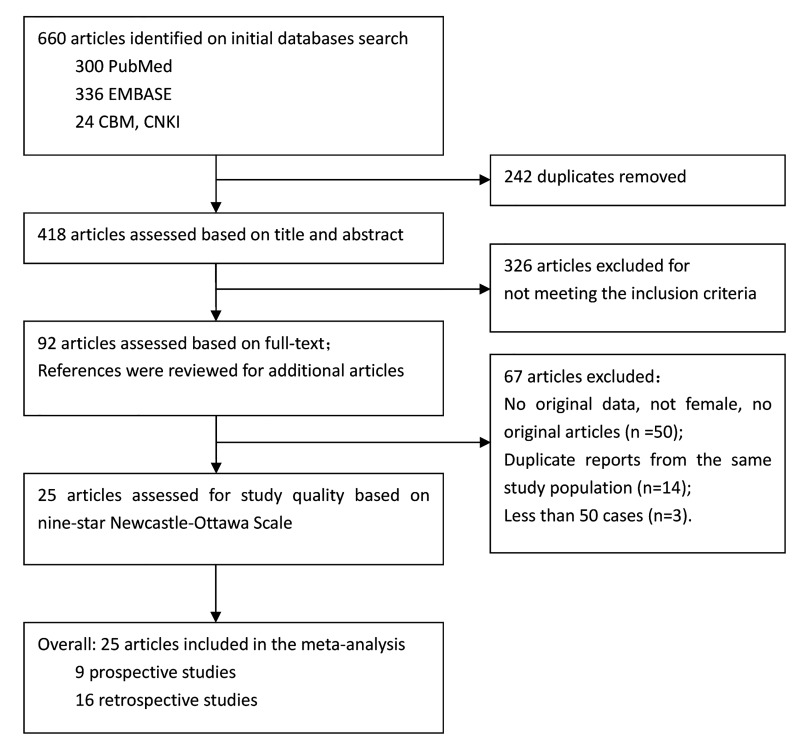
Flowchart for study selection.

Studies were included for statistical analyses if they fulfilled the following criteria: to be observational studies (case-control or cohort studies); to have measured PCB levels in biological samples (adipose tissue, serum or plasma); to have reported measures of association (odds ratio, relative ratio) and 95% confidence intervals (95% CIs) for breast cancer risk. We excluded studies which had no biomarker data; no original data or observations; fewer than 50 breast cancer cases; study subjects were not female; articles were reviews, letters to the editor, editorial reports, case reports, duplicate publications, or abstracts. When there were several articles about the same cohort, the article reporting the largest number of cases was selected. The procedure of study selection is summarized in Fig 1.

### Data Extraction

The following variables were recorded using data abstraction forms: First author, year of publication, country of study, ages of participants, study period, sample size, study design, specimen type, number of congeners measured, mean exposure level of total PCBs, and variables adjusted for in the analysis. To summarize the main results, we have chosen the measures of total PCB exposure and selected PCB congeners classified as group I (potentially estrogenic), group II (potentially antiestrogenic and immunotoxic, dioxin-like), and group III (phenobarbital, CYP1A and CYP2B inducers, biologically persistent) according to their structural, biological and pharmacokinetics properties proposed by Wolff and Toniolo [16, 24]. Group I includes major PCB congeners 44, 49, 52, 101, 174, 177, 187 and 201; group II includes major PCB congeners 66, 74, 77, 105, 118, 126, 128, 138, 156, 167, 169 and 170; group III includes major PCB congeners 99, 153, 180, 183, 196 and 203 [16, 24]. Since the articles reported PCB exposure for 3–5 levels, the most fully adjusted OR with corresponding 95% CIs for the highest versus lowest categories were selected in this meta-analysis. The main findings of the selected studies are presented in Table 1 and Table 2.

**Table 1:** Characteristics of prospective epidemiologic (nested case-control) studies on PCB exposure and breast cancer risk.

Study	Location	Study Period	No. of cases: controls	Age	No. of measured congeners	Mean±SD of PCBs (ng/g lipid)		Adjustment for Covariates	Biologic Specimen	Study Quality ^d^
						Cases	Controls			
Laden et al., 2001	USA	1976–1994	370:370	43–69	21	544 ^b^	543 ^b^	family history of breast cancer, history of BBD, age at menarche, BMI, lipid, number of children, age at birth of first child, lactation.	plasma	8
Ward et al., 2000	Norway	1973–1991	150:150	18–60	26	776.1	806.6	lipid concentration in serum.	serum	7
Wolff et al., 2000	USA	1985–1994	148:295	34–65	NR	683 (1.64) ^c^	663 (1.62) ^c^	family history of breast cancer, age at menarche, number of children, age at birth of first child, lipid, lactation.	serum	9
Helzlsouer et al., 1999	USA	1974–1994	235:235	35–70	26	735.3±644.8	663.6±322.5	lipid concentration in serum.	serum	8
Krieger et al., 1994	USA	1964–1990	150:150	45.2(9.6) Mean(SD)	NR	4.4±1.8 ng/ml ^a^	4.8±2.5 ng/ml ^a^	BMI, age at menarche, pregnant, menopausal status, race, year of joining program (±1), age and year of multiphasic examination, follow-up time.	serum	8
Dorgan et al., 1999	USA	1977–1987	105:208	47–67	27	NR	NR	age, history of BBD, lipid, year of blood collection.	serum	8
Cohn et al., 2012	USA	1959–1998	112:112	<50	16	0.50 mmol/l ^a^ ^,^ ^b^	0.43 mmol/l ^a^ ^,^ ^b^	cholesterol, triglycerides, race, parity, lactation, BMI, year of blood collection.	serum	7
Høyer et al., 1998	Denmark	1976–1995	240:477	>20	28	1099.89 (547.59) ^b^		age, weight, height, number of children, alcohol, smoking, lipid, physical activity, menopausal status, household income, marital status, education.	serum	8
Raaschou-Nielsen et al., 2005	Denmark	1993–1997	409:409	50–64	18	NR	NR	education, BMI, lipid, alcohol, number of children, age at birth of first child, lactation, HRT, history of BBD.	adipose tissue	9

Abbreviations: SD, standard deviation; BBD, benign breast disease; BMI, body mass index; HRT, hormone replacement therapy; NR, not reported.

a: Not lipid-adjusted serum concentrations.

b: median and/or interquartile range

c: geometric mean and/or standard deviation

d: Study quality was judged on the basis of the Newcastle-Ottawa Scale (1–9 stars).

**Table 2:** Characteristics of retrospective epidemiologic (case-control) studies on PCB exposure and breast cancer risk.

Study	Location	Study Period	No. of cases: controls	Age	No. of measured congeners	Mean±SD of PCBs (ng/g lipid)		Adjustment for Covariates	Biologic Specimen	Study Quality ^d^
						Cases	Controls			
Zhang et al., 2013	China	2010–2011	92: 92 (H)	27–68	7	4.5897 ng/ml ^a^ ^,^ ^b^	2.7683 ng/ml ^a^ ^,^ ^b^	family history of breast cancer, history of BBD, age at menarche, lactation, menstrual cycle, time between menarche and primiparity, bean products intake.	serum	6
Itoh et al., 2009	Japan	2001–2005	403:403 (H)	20–74	41	170 (120–220) ^b^	180 (140–240) ^b^	age, area, BMI, lipid, menopause, smoking, fish and vegetable consumption, family history of breast cancer, age at first child birth, age at menarche, history of breast cancer screening, lactation.	serum	7
Gatto et al., 2007	USA	1995–1998	355:327 (P)	35–64	Aroclors 1242, 1260	310±310	310±340	age, BMI, lactation, lipid.	serum	7
Rubin et al., 2006	USA	1983–1987	63: 63 (P)	>45	28	5.30 ng/ml ^b^	8.08 ng/ml ^b^	parity, family history of breast cancer, race, triglycerides, cholesterol.	serum	7
Charlier et al., 2004	Belgium	NR	60: 60 (P)	48–61	5	7.08±7.51 ng/ml ^a^	5.10±5.15 ng/ml ^a^	age, age at menarche, menopause, HRT, parity, lactation, family history of breast cancer.	serum	6
Lopez-Carrillo et al., 2002	Mexico	1994–1996	95: 95 (H)	20–79	Aroclors 1260	833 (26–20010.2)^b^	833 (26–6078.1)^b^	age at menarche, number of children, age at first birth, lactation, lipid, family history of breast cancer, menopausal status, quetelet index.	serum	6
Zheng et al., 2000	USA	1995–1997	475: 502 (H, BBD)	30–80	9	733.1 (706.3–761.0) ng/ml ^c^	747.6 (721.0–775.1) ng/ml ^c^	BMI, lipid, age at menarche, number of children, age at birth of first child, lactation, HRT, fat intake, family breast cancer history, income, race, study site.	serum	7
Moysich et al., 1998	USA	1986–1991	154:192 (P)	41–85	73	4.29±2.40	4.12±2.24	age, education, family history of breast cancer, parity, quetelet index, lactation, age at first birth, years since last pregnancy, fruit and vegetable intake, lipid.	serum	8
Gammon et al., 2002	USA	1996–1997	638:423 (P)	24–96	24	386.72 (1.69) ^c^	391.74 (1.74) ^c^	age, race, lipid, history of fertility problems, history of BBD.	serum	8
Wolff et al., 2000	USA	1994–1996	175: 355 (H, BBD)	56 (13) ^b^	14 (HPCB)	600 (1.88) ^c^	620 (1.86) ^c^	age, age-squared, menopausal status, race, BMI, lipid, family history of breast cancer, lactation, parity.	serum	6
Ye et al., 2009	China	2005–2007	78: 72 (P)	40–64	7	1.397 (0.047–13.125) ng/ml ^a^ ^,^ ^b^	0.788 (0.096–16.082) ng/ml ^a^ ^,^ ^b^	age, BBD, lactation.	serum	6
Millikan et al., 2000	USA	1993–1996	748: 659 (P)	21–74	35	NR	NR	age, age-squared, race (all participants), lipid, menopausal status, BMI, parity/lactation, HRT, income.	plasma	7
Demers et al., 2002	Canada	1994–1997	314: 523 (H, P)	30–70	14	NR	NR	age, region of residence, BMI, lipid, personal history of BBD, lactation.	plasma	6
Stellman et al., 2000	USA	1994–1996	232: 323 (H, BBD)	<82	14	294.7	257.1	age, BMI, lipid, hospital, race.	adipose tissue	6
Aronson et al., 2000	Canada	1995–1997	217:213 (H, BBD)	<80	14	940 (880–1000) ^c^	870 (810–920) ^c^	HRT, race, lipid, family history, fat intake, alcohol.	adipose tissue	6
Recio-Vega et al., 2011	Mexico	NR	70: 70 (H)	25–80	20	5.26 (3.50–7.90) ng/ml ^a^ ^,^ ^c^	3.33 (2.37–4.67) ng/ml ^a^ ^,^ ^c^	age, age at menarche, lactation, menopausal status, BMI.	serum	5

Abbreviations: SD, standard deviation; H, hospital control group; P, population control group; BBD, benign breast disease; BMI, body mass index; HRT, hormone replacement therapy; NR, not reported.

a: Not lipid-adjusted serum concentrations.

b: median and/or interquartile range

c: geometric mean and/or standard deviation

d: Study quality was judged on the basis of the Newcastle-Ottawa Scale (1–9 stars).

### Study quality assessment

We assessed study quality by using the nine-star Newcastle-Ottawa Scale (NOS) [25]. The NOS has three dimensions including Selection, Comparability, and Outcome (cohort studies) or Exposure (case-control studies). Response options are provided for each item. A star system (zero to nine stars), is used to allow a semi-quantitative assessment of study quality. Within the Selection and Outcome-Exposure categories, a study can be awarded a maximum of one star for each item. For Comparability, a maximum of two stars can be given. If the study adjusted lipid as the main confounder, we gave one star; if the study adjusted one of the additional confounders like age, body mass index (BMI), lactation, family history of breast cancer or age at menarche, we gave another one star. High-quality studies were defined as having scores ≥8, and moderate quality, scores 5 to 7.

### Statistical Analysis

The pooled results were reported as ORs with 95% CIs. For one study [26] that reported results separately for five individual PCB congeners, category-specific ORs, and variances were combined using a fixed-effects model based on inverse variance weight to obtain combined estimates for total PCBs, before estimating the study-specific OR and 95% CI. Statistical heterogeneity in the relationship between PCB exposure and breast cancer among studies was evaluated with Q and *I* ^2^ statistics [27, 28]. *I* ^2^ describes the percentage of total variation across studies that is due to heterogeneity rather than chance, which quantifies the effect of heterogeneity of the studies. The range is defined as: *I* ^2^<25%: low heterogeneity; *I* ^*2*^ 25–50%: moderate heterogeneity; *I* ^*2*^>50% high heterogeneity [29]. Study-specific individual OR estimates were combined based on a fixed-effects model. When heterogeneity was moderate we used a DerSimonian-Laird random-effects model, which considers both within-study and between-study variation [30]. When heterogeneity was high, subgroup analyses were conducted by *a priori* selected variables (study design; type of biological specimen: serum, plasma, or adipose tissue; study location) related to potential effect modifiers to identify sources of heterogeneity.

To explore the stability of the results, several sensitivity analyses were performed, including the influence analysis, in which one study in the meta-analysis was dropped at a time to determine the influence of the individual dataset on the pooled ORs, and meta-analysis excluded the studies which might bring high heterogeneity. Publication bias was assessed visually using funnel plots and statistically using Begg’s test and Egger’s test [31, 32]. Statistical significance was considered at P<0.05, two-sided. All statistical analyses were performed using Stata 12 software (Stata Corp, College Station, TX).

## Results

### Literature Search

The initial search yielded 660 citations (242 of which were duplicates) from four different databases. After screening the 418 nonduplicated articles based on title and abstract, 92 articles appeared to be potentially relevant for doing full-text screening. We excluded 67 articles for the following reasons: duplicate reports from the same study population (n = 14); articles reporting fewer than 50 breast cancer cases (n = 3); no original data or subjects were not female or articles were editorials, comments, case reports or reviews (n = 50); The remaining 25 articles were evaluated in the study quality assessment step before meta-analysis (Fig 1).

### Study quality

Study quality was assessed based on scoring using the Newcastle-Ottawa Scale, categorized into three dimensions: Selection, Comparability, and Outcome or Exposure. The qualities of the retained 25 studies were moderate to high (5 to 9 stars); the prospective studies met higher quality criteria (7 to 9 stars) than the retrospective studies (5 to 8 stars). The methods and reported data of all included studies were judged to be adequate. The evaluation of quality of included studies is presented in S4 Table. Six studies did not adjust the lipid. The quality of prospective epidemiologic (nested case-control) studies was mainly compromised by the low representativeness of the exposed cohort. For the case-control studies, their quality was often compromised by the lack of description for ascertainment of PCB exposure as well as the potential case selection biases and use of hospital controls.

### Study Characteristics

A total of 6088 cases in 25 studies published between 1994 and 2013 were analyzed. Of the 25 studies, 14 were conducted in the United States [33–46], two in Canada [47, 48], two in China [49, 50], two in Denmark [51, 52], two in Mexico [53, 54], one in Norway [55], one in Japan [56], and one in Belgium [26]. Nine nested case-control prospective studies included 1919 cases [33–38, 51, 52, 55], the remaining 16 case-control retrospective studies included 4169 cases [26, 39–50, 53, 54, 56]. Most studies provided risk estimates adjusted for age, body mass index, family history of breast cancer, history of benign breast disease, age at menarche, and lactation. Some studies were adjusted for menopausal status, alcohol, smoking, fish and vegetable consumption, race, or income (Table 1, Table 2).

### Associations between PCB exposure and breast cancer

**Fig 2:**
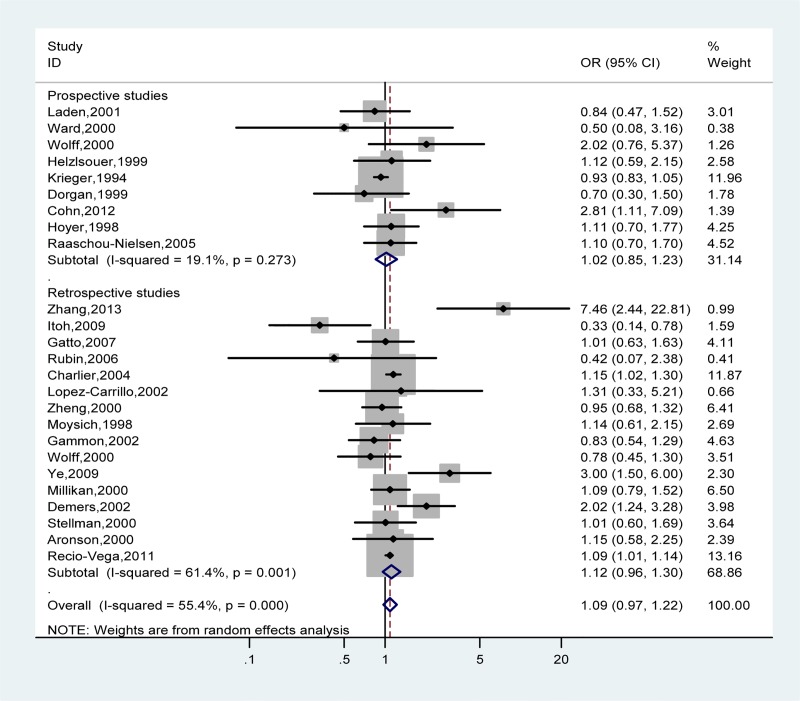
Forest plot describing the association between total PCB exposure and breast cancer risk. Apart from the overall analysis, the subgroup analyses on prospective (upper panels) and retrospective (lower panels) studies are presented.

The multivariable-adjusted OR for each study and combination of all studies stratified by study design for the highest versus lowest categories of total PCB exposure and breast cancer risk is shown in Fig 2. Overall, the summary OR of total PCBs was slightly elevated but not statistically significant and heterogeneity was relatively high (OR = 1.09, 95%CI: 0.97–1.22, *I* ^2^ = 55.4%). There was low heterogeneity among prospective studies (*I* ^2^ = 19.1%), but among retrospective studies the heterogeneity was high (*I* ^2^ = 61.4%).

**Fig 3:**
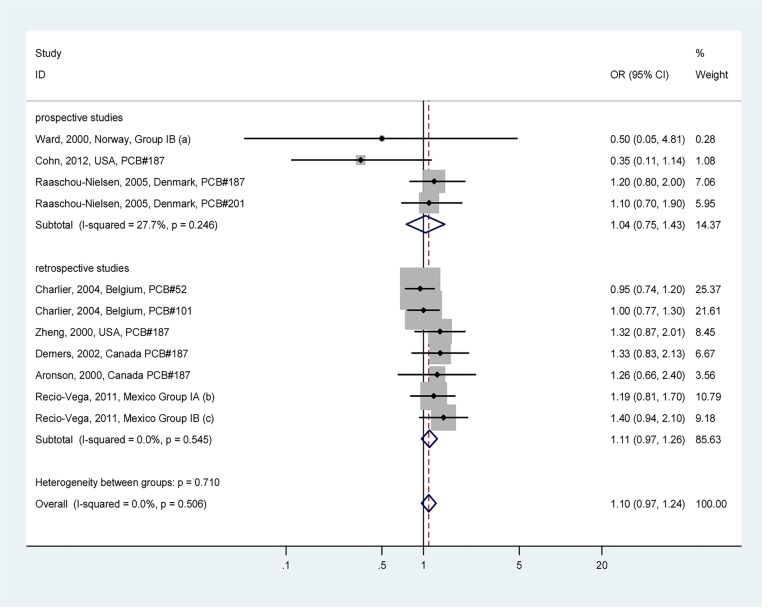
Forest plot describing the association between potentially estrogenic PCBs (Group I) exposure and breast cancer risk. Apart from the overall analysis, the subgroup analyses on prospective (upper panels) and retrospective (lower panels) studies are presented. (a) Group IB includes PCB congeners 177, 187 and 201; (b) Group IA includes PCB congeners 44, 52; (c) Group IB includes congeners 101, 187.

**Fig 4:**
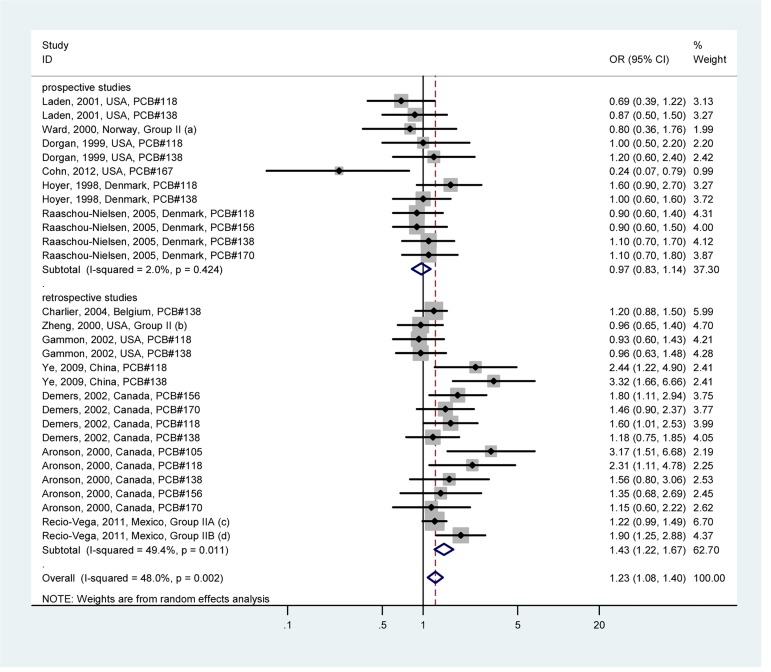
Forest plot describing the association between potentially antiestrogenic and immunotoxic, dioxin-like PCBs (Group II) exposure and breast cancer risk. Apart from the overall analysis, the subgroup analyses on prospective (upper panels) and retrospective (lower panels) studies are presented. (a) Group II includes PCB congeners 74, 118, 138, 156 and 170; (b) Group II includes PCB congeners 74, 118, 138, 156, and 170; (c) Group IIA includes congeners 66, 77, 105, 118 and 126; (d) Group IIB includes congeners 128, 138 and 170.

**Fig 5:**
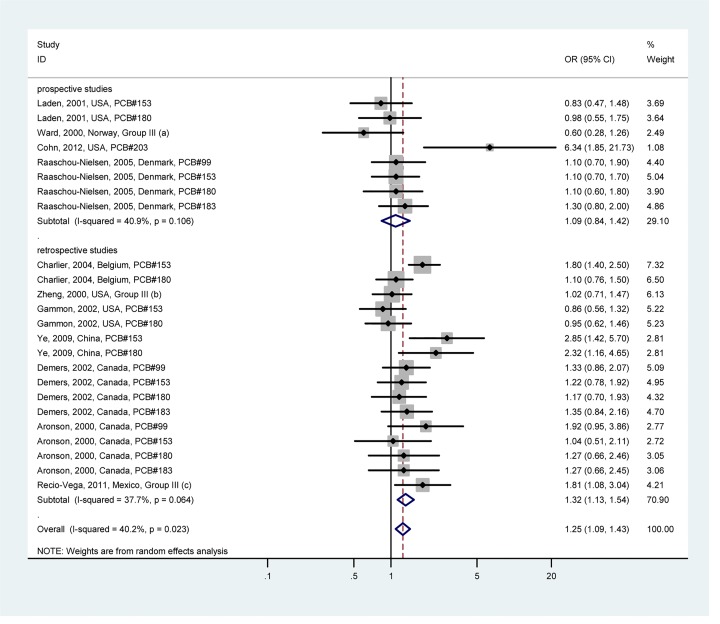
Forest plot describing the association between phenobarbital, CYP1A and CYP2B inducers, biologically persistent PCBs (Group III) exposure and breast cancer risk. Apart from the overall analysis, the subgroup analyses on prospective (upper panels) and retrospective (lower panels) studies are presented. (a) Group III includes 153, 180 and 183; (b) Group III includes 153, 180, and 183; (c) Group III includes congeners 153 and 180.

Potentially estrogenic PCBs (Group I) exposure and breast cancer risk are shown in Fig 3; potentially antiestrogenic and immunotoxic, dioxin-like PCBs (Group II) exposure and breast cancer risk are shown in Fig 4; phenobarbital, CYP1A and CYP2B inducers, biologically persistent PCBs (Group III) exposure and breast cancer risk are shown in Fig 5. For potentially estrogenic PCBs (Group I), the association with breast cancer was not significant (OR = 1.10, 95%CI: 0.97–1.24). For group II and group III, the associations with breast cancer were significant, and the heterogeneity was acceptable (*I* ^*2*^ = 48.0% and *I* ^*2*^ = 40.2%, respectively). The pooled ORs were 1.23 (95%CI: 1.08–1.40) for group II and 1.25 (95%CI: 1.09–1.43) for group III, respectively. Summary of associations between PCB exposure and breast cancer risk are presented in S3 Table.

### Subgroup analyses for retrospective studies of total PCBs meta-analysis

Subgroup analyses were performed by specimen types and study locations to further explore the sources of the observed high heterogeneity in retrospective studies of total PCBs (Table 3). After stratification by the subgroups, the high degree of heterogeneity still could not be resolved, but the results showed that the heterogeneity was mainly from the studies which used serum specimens or were conducted in Asia. For the North America and Europe subgroups, there appeared statistically significant associations with breast cancer risk and the heterogeneity was low, however, the association was still slightly elevated.

**Table 3:** Subgroup analyses for retrospective studies of total PCBs.

Subgroups	Number of studies	OR (95% CI)	Heterogeneity	
			*I* ^2^ (%)	P
Specimen type				
Serum/plasma	14	1.12 (0.95, 1.32)	66.5	<0.001
Adipose tissue	2	1.06 (0.70, 1.60)	0.0	0.765
Geographical location				
North America	12	1.08 (1.01, 1.16)	2.2	0.423
Asia	3	1.91 (0.34, 10.68)	91.4	<0.001
Europe	1	1.15 (1.02, 1.30)	NA	NA

Abbreviations: NA, not applicable.

### Sensitivity Analysis

Several sensitivity analyses were performed to explore the stability of the results. After one study was removed at a time, we found that the influence of each individual data set on the pooled ORs was not significant (S1–S4 Figs). For the total PCBsn in a meta-analysis excluding three retrospective studies [49, 50, 56], with divergent odds ratios (Ye [49], OR = 3.00; Zhang et al [50], OR = 7.46; Itoh et al [56], OR = 0.33), the association was slightly attenuated but still not statistically significant (OR = 1.06, 95% CI: 0.98–1.15, *I* ^2^ = 20.7%, P = 0.188) (S5 Fig).

### Publication Bias

Begg’s test [31] and Egger’s test [32] did not find publication bias among the studies. For total PCBs (Begg’s P = 0.498, Egger’s P = 0.668), for group I (Begg’s P = 0.640, Egger’s P = 0.814), for group II (Begg’s P = 0.302, Egger’s P = 0.658), for group III (Begg’s P = 0.244, Egger’s P = 0.432).

## Discussion

We conducted a meta-analysis to evaluate the evidence from epidemiologic studies on PCB congener exposure and the risk of breast cancer. The results showed that the risk of breast cancer was associated with exposure to group II (potentially antiestrogenic and immunotoxic, dioxin-like) and group III PCBs (phenobarbital, CYP1A and CYP2B inducers, biologically persistent) exposure, but not with group I (potentially estrogenic) or total PCB exposure.

In 2013, Zani et al. conducted a meta-analysis of epidemiological studies on cancer risks for exposure to PCBs. Like us, they found no significant association between breast cancer risk and total PCB exposure for highest versus lowest categories (OR = 1.15, 95% CI: 0.92–1.43, *I* ^*2*^ = 70.6%) [57]. Our study included the articles with the largest number of cases from the same study populations as Zani’s and added two more studies conducted in China which met our selection criteria [49, 50]. In addition, since the amounts and proportions of PCB congeners do vary widely from individual to individual, we further explored the relationships with the different PCB congeners grouped by functional significance. Many of the studies included in our meta-analysis did not conduct congener-specific analyses due to some limitations. We identified eight studies for group I congeners, 13 studies for group II, and 11 studies for group III. The selected PCB congeners and ORs of three PCB groups are presented in S2 Table. To our knowledge, ours is the first study to quantitatively evaluate the association between PCB congener groups and breast cancer risk, and the results indicate that high exposure to group II and group III PCBs can increase the risk of breast cancer.

For group I PCBs, one suggestion is that the low-persistence PCB congeners may not well reflect the exposure levels at the time of the PCB insult, but rather more recent changes in exposure and intake in breast cancer cases, such as dietary habit change after the diagnosis [58]. For group II PCBs, Wolff et al. stated that the antiestrogenic effects of the PCB congeners could lead to protection against breast cancer [16], which seems to run contrary to current findings. However, some studies also proposed that the dioxin-like effects of the PCB congeners in group II might induce cell differentiation [59], as well as the biotransformation enzymes which in turn affect estradiol metabolism that may increase breast cancer risk [60]. Group III PCBs may influence estradiol metabolism to the more toxic 16-α-hydroxy estradiol and enhance metabolism of carcinogens, and some congeners in this category may also act as hormonal agonists [61]. Some studies showed that CYP1A and CYP1B1 enzyme induction increases the formation of 4-hydroxy-estradiol, a catechol estrogen that damages DNA through the formation of reactive free radicals [62]. Associations between serum levels of POPs, genetic polymorphisms and breast cancer has been observed as well [63, 64]. There is some overlap in the ability of PCB congeners to induce enzyme activity. Group III congeners may induce the CYPlAl group to some extent and CYP2A1 may be induced by both group II and III congeners [9]. In addition, groups II and III are classified as heavy congeners, which are associated with disease more frequently, due to their slow metabolism and persistence in the body [53].

For total PCBs, most studies conducted in North America or Europe showed null results, although current levels are likely to be indicative of past exposures because of the long half-lives of the compounds and their resistance to metabolism, years after exposure, the evaluation of risks associated with low levels from a general population sample still could be biased to the null due to non-differential measurement error [33]. Another potential limitation may be that, differences in correlations among PCBs, body mass index (BMI) and PCB half-lives denote different intervals of exposure and rates of elimination across the population. BMI may be a significant factor, causing marked disparities in circulating PCB levels between lean and obese women and leading to inter-individual variations that are not proportional over time. Therefore, although a measurement of persistent PCB levels in the body may reflect lifetime exposure, a single PCB measurement may not accurately represent the past exposure of a population or characterize PCB levels at a time that is relevant to cancer [34]. Since PCBs have shown decreasing trends after the production and use bans came into effect [65, 17, 66], the different study time periods could have an unintended effect on the results. In our study, for nested case-control studies, most study periods were around the years when PCBs were banned, and they depended on the pre-existence of cohorts that have been followed over time. The information on exposure and sample taken were all collected at baseline before the outcome of interest occurred, and the most appropriate control group was chosen from members of the same cohort who had not developed breast cancer at the time they were chosen. These advantages of the prospective studies may overcome some potential bias in retrospective case control studies, so they could reflect the association between exposure levels and breast cancer risk more adequately. Most case-control studies were conducted many years after PCBs were banned, so they mostly reflect PCB exposure in the past. The long latency could make the time of clinical diagnosis different from the time when breast cancer began to develop, and having control groups from different populations such as communities or hospitals can also bring heterogeneity.

Overall, we observed slightly elevated levels of heterogeneity among the total PCB exposure studies (*I* ^2^ = 55.4%). After stratification by study design, the heterogeneity among prospective studies was low, but among retrospective studies the heterogeneity persisted high. After further stratifying the retrospective studies by specimen-source subgroups, the heterogeneity did not disappear. For the specimen type subgroup, one possible explanation for the heterogeneity is that serum levels can be influenced by factors that relate to the outcome of interest, such as the weight loss experienced by cancer patients at advanced stages, which can mobilize the PCBs stored in the adipose tissue and then increase the serum PCB levels. For the study site subgroup, the heterogeneity among studies in Asia (two in China and one in Japan) was extremely high. However, the North America and European retrospective studies indicated a borderline statistically significant risk for breast cancer associated with PCB exposure and there appeared low heterogeneity among the studies.

There are also several limitations of this meta-analysis. First, one of the included studies conducted by Ye [49] is an unpublished thesis. However, the quality of the study was judged to be adequate (the Newcastle-Ottawa-Scale score was 6). Sensitivity analysis showed this study did not affect the pooled ORs significantly (S1–S4 Figs). We also did meta-analysis without the Ye study, and the results were not changed (data not shown). Second, although most studies adjusted for the common risk factors for breast cancer, there are six studies did not adjust lipid as a main confounder and unknown confounders can’t be excluded as potential explanations for the observed findings. In addition, the definite dose for PCB exposure differed slightly across the studies and the different PCB exposure measurements used in different studies may also bring heterogeneity. Third, it is possible that exposure to mixtures of PCBs and other chemicals with estrogenic properties and other organochlorine pesticides may also affect breast cancer risk. Some in vitro studies suggest that mixtures of complex organochlorine compounds can increase the proliferation of MCF-7 cells due to their estrogenic potential [67, 68]. Other studies have shown that the three PCBs most abundant in biological extracts, 2,2',3'4,4',5-hexachlorobiphenyl (PCB138), 2,2',4,4',5,5'-hexachlorobiphenyl (PCB153), and 2,2',3,4,4',5,5'-heptachlorobiphenyl (PCB180) (the single compounds and their mixtures) have pleiotropic effects on the estrogen and androgen receptor. Thus any simple evaluation of the chemical level of the estrogenic, antiestrogenic interaction is complex as the compounds are found in the blood together [69]. Interestingly, studies have extracted the fraction containing the mixture of legacy POPs, including the PCBs, and found a correlation between the chemical level of POP biomarkers and the xenoestrogenicity of the serum POP mixture related to the detected level: the xenoestrogenic activity of the Inuit's (high serum PCB levels) and Warsaw study groups (lower PCB levels) elicited high frequency of samples with ER antagonistic and agonistic activity, respectively. It was suggested that the variation in xenoestrogenic serum activity reflects differences in POP exposure mixture, genetic factors and/or life style factors [70]. Moreover, the xenoestrogenicity and xenoandrogenicity of the extracted fraction of serum legacy POP /PCBs was found to be higher in breast cancer cases, however, only the xenoandrogenicity was significantly related to the risk of breast cancer [71]. Thus, there is a need for further investigation to explore the relationship between mixtures of organochlorine compounds and breast cancer.

Our meta-analysis has several advantages. First, to the best of our knowledge, this is the first meta-analysis that included the studies conducted in China as a developing country, notably, the Chinese studies showing statistically significant strong risk for breast cancer associated with PCB exposure. There are huge differences in amount and mode of exposure between developing and developed countries and there is a dearth of studies to assess the risk of breast cancer in developing countries, which can’t be made up for by generalizing the results from developed countries [72]. Second, we excluded studies [71, 58, 73] based on fewer than 50 breast cancer cases to minimize small study effects. In these studies, two of them, separately conducted by Liljegren et al. [73] and Bonefeld-Jorgensen et al. [71], found no significant higher risk of breast cancer with total PCB exposure. However, one study conducted by Pavuk et al. [58] found higher serum levels (highest vs. lowest tertile) of total PCBs and PCB congener groups classified by Wolff et al. [24] were inversely associated with risk of breast cancer (S5 Table). Third, the quality of included studies was judged to be adequate and the tests did not identify publication bias, which indicates that the pooled result’s bias may be negligible.

## Conclusion

In summary, the results of our meta-analysis based on the observational studies found that the risk of breast cancer was associated with group II and group III PCB exposure, but not with group I or total PCB exposure. Still, more studies in developing countries, and further studies to explore the relationship between mixtures of organochlorine compounds and the risk of breast cancer are needed.

## Supporting Information

S1 PRISMA Checklist PRISMA Checklist. (DOC)

S1 Fig The result of influence analysis for total PCBs. (TIF)

S2 Fig The result of influence analysis for group I PCBs. (a) Group IB includes PCB congeners 177, 187 and 201; (b) Group IA includes PCB congeners 44,52; (c) Group IB includes congeners 101,187.
(TIF)

S3 Fig The result of influence analysis for group II PCBs. (a) Group II includes 74,118,138, 156 and 170; (b) Group II includes PCB congeners 74,118,138,156, and 170; (c) Group IIA includes congeners 66,77,105,118 and 126; (d) Group IIB includes congeners 128,138 and170.
(TIF)

S4 Fig The result of influence analysis for group III PCBs. (a) Group III includes 153,180 and 183; (b) Group III includes congeners 153 and 180.
(TIF)

S5 Fig Forest plot of sensitivity analysis describing the association between total PCB exposure and breast cancer risk excluding three retrospective studies. Apart from the overall analysis, the subgroup analyses on prospective (upper panels) and retrospective (lower panels) studies are presented.
(TIF)

S1 Table Database search strategy. (DOC)

S2 Table Association between breast cancer risk and PCB congeners grouped according to the classification proposed by Wolff. (DOC)

S3 Table Association between PCB exposure and breast cancer risk. (DOC)

S4 Table Evaluation of quality based on the Newcastle-Ottawa scale for the included studies. (DOC)

S5 Table Characteristics of the excluded studies based on less than 50 breast cancer cases. (DOC)
