# Supplementary figures and images for: Environmental Polychlorinated Biphenyl Exposure and Breast Cancer Risk: A Meta-Analysis of Observational Studies
Source: PLoS One. 2015 Nov 10;10(11):e0142513. doi: 10.1371/journal.pone.0142513 (PMC4640539; doi:10.1371/journal.pone.0142513)

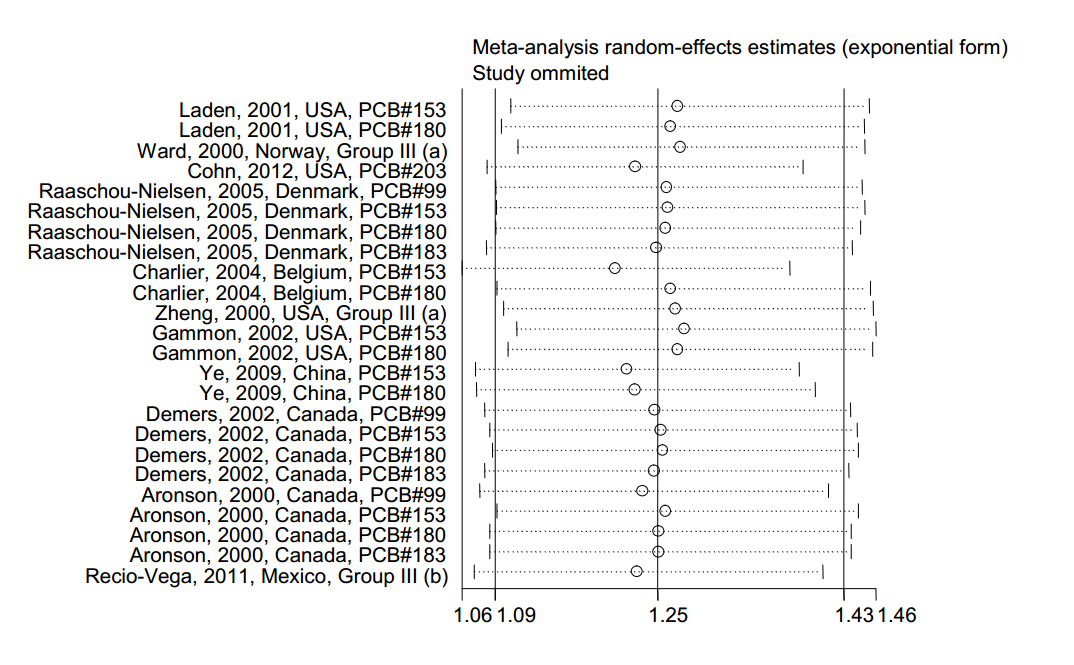

Supplement: S4 Fig — (a) Group III includes 153,180 and 183; (b) Group III includes congeners 153 and 180. (TIF) [file pone.0142513.s005.tif]

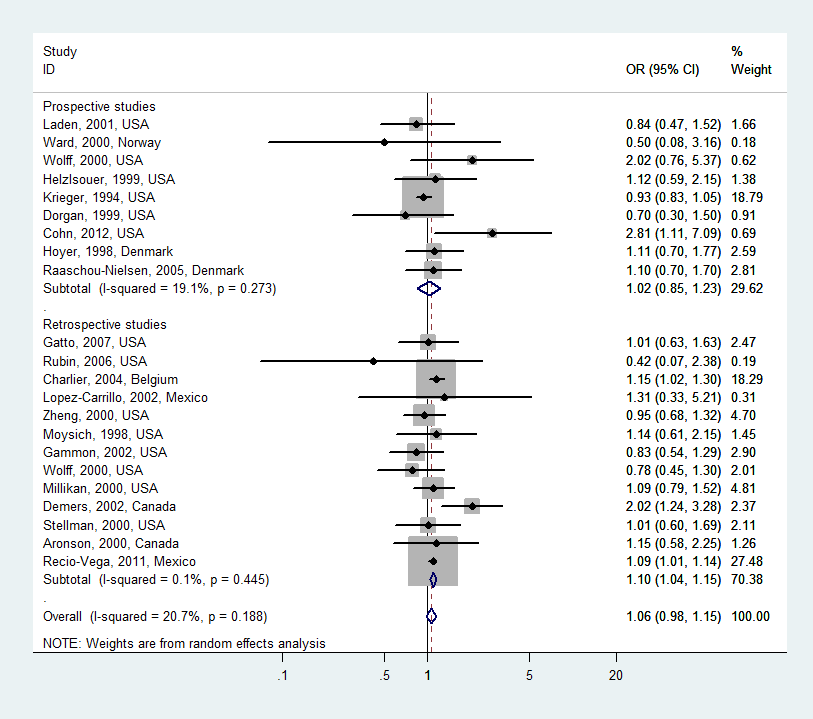

Supplement: S5 Fig — Apart from the overall analysis, the subgroup analyses on prospective (upper panels) and retrospective (lower panels) studies are presented. (TIF) [file pone.0142513.s006.tif]
