# Supplementary material for: Environmental Polychlorinated Biphenyl Exposure and Breast Cancer Risk: A Meta-Analysis of Observational Studies
Source: PLoS One. 2015 Nov 10;10(11):e0142513. doi: 10.1371/journal.pone.0142513 (PMC4640539; doi:10.1371/journal.pone.0142513)
Supplement: S1 Table — (DOC) [file pone.0142513.s007.doc]

**S1 Table. Database search strategy**

| PubMed | (("Polychlorinated Biphenyls"[mh] OR polychlorinated biphenyls OR polychlorobiphenyl OR biphenyls, polychlorinated OR polychlorobiphenyl compounds OR compounds, polychlorobiphenyl OR PCB*) AND (breast neoplasms [mh] OR ( (breast OR mammary) AND (cancer OR neoplasm* OR carcinoma* OR tumor OR tumour*)))) |
| --- | --- |
| Embase | ((Polychlorinated Biphenyls OR polychlorinated biphenyls OR polychlorobiphenyl OR biphenyls, polychlorinated OR polychlorobiphenyl compounds OR compounds, polychlorobiphenyl OR PCB*) AND (breast cancer OR ( (breast OR mammary) AND (cancer OR neoplasm* OR carcinoma* OR tumor OR tumour*)))) |
