# Supplementary material for: Environmental Polychlorinated Biphenyl Exposure and Breast Cancer Risk: A Meta-Analysis of Observational Studies
Source: PLoS One. 2015 Nov 10;10(11):e0142513. doi: 10.1371/journal.pone.0142513 (PMC4640539; doi:10.1371/journal.pone.0142513)
Supplement: S2 Table — (DOC) [file pone.0142513.s008.doc]

**S2 Table. Association between breast cancer risk and PCB congeners grouped according to the classification proposed by Wolff.**

Abbreviations: OR, odds ratio; 95%CI, 95% conﬁdence interval; NR, not reported.

| Study | | group of PCB congeners | | | | | |
| --- | --- | --- | --- | --- | --- | --- | --- |
|  |  | I | | II | | III | |
|  |  | Congener(s) | OR(95%CI) | Congener(s) | OR(95%CI) | Congener(s) | OR(95%CI) |
|  | |  |  |  |  |  |  |
| Prospective epidemiologic (nested case-control) studies | | | | | | | |
| Laden et al.,2001 | NR | | NR | #118 | 0.69(0.39,1.22) | #153 | 0.83(0.47,1.48) |
|  |  | |  | #138 | 0.87(0.50,1.50) | #180 | 0.98(0.55,1.75) |
| Ward et al.,2000 | Group IB^a^ | | 0.50(0.05,4.81) | Group II | 0.80(0.36,1.76) | Group III | 0.60(0.28,1.26) |
| Dorgan et al.,1999 | NR | | NR | #118 | 1.00(0.50,2.20) | NR | NR |
|  |  | |  | #138 | 1.20(0.60,2.40) |  |  |
| Cohn et al.,2012 | #187 | | 0.35(0.11,1.14) | #167 | 0.24(0.07,0.79) | #203 | 6.34(1.85,21.73) |
| Høyer et al.,1998 | NR | | NR | #118 | 1.60(0.90,2.70) | NR | NR |
|  |  | |  | #138 | 1.00(0.60,1.60) |  |  |
| Raaschou-Nielsen  et al.,2005 | #187 | | 1.20(0.80,2.00) | #118 | 0.90(0.60,1.40) | #99 | 1.10(0.70,1.90) |
|  | #201 | | 1.10(0.70,1.90) | #156 | 0.90(0.60,1.50) | #153 | 1.10(0.70,1.70) |
|  |  | |  | #138 | 1.10(0.70,1.70) | #180 | 1.10(0.60,1.80) |
|  |  | |  | #170 | 1.10(0.70,1.80) | #183 | 1.30(0.80,2.00) |
| Retrospective epidemiologic (case-control) studies | | | | | | | |
| Charlier et al.,2004 | | #52 | 0.95(0.74,1.20) | #138 | 1.20(0.88,1.50) | #153 | 1.80(1.40,2.50) |
|  |  | #101 | 1.00(0.77,1.30) |  |  | #180 | 1.10(0.76,1.50) |
| Zheng et al.,2000 | | #187 | 1.32(0.87,2.01) | Group II^b^ | 0.96(0.65,1.40) | Group III | 1.02(0.71,1.47) |
| Gammon et al.,2002 | | NR | NR | #118 | 0.93(0.60,1.43) | #153 | 0.86(0.56,1.32) |
|  |  |  |  | #138 | 0.96(0.63,1.48) | #180 | 0.95(0.62,1.46) |
| Ye et al.,2009 | | NR | NR | #118 | 2.44(1.22,4.90) | #153 | 2.85(1.42,5.70) |
|  |  |  |  | #138 | 3.32(1.66,6.66) | #180 | 2.32(1.16,4.65) |
| Demers et al.,2002 | | #187 | 1.33(0.83,2.13) | #156 | 1.80(1.11,2.94) | #99 | 1.33(0.86,2.07) |
|  |  |  |  | #170 | 1.46(0.90,2.37) | #153 | 1.22(0.78,1.92) |
|  |  |  |  | #118 | 1.60(1.01,2.53) | #180 | 1.17(0.70,1.93) |
|  |  |  |  | #138 | 1.18(0.75,1.85) | #183 | 1.35(0.84,2.16) |
| Aronson et al.,2000 | | #187 | 1.26(0.66,2.40) | #105 | 3.17(1.51,6.68) | #99 | 1.92(0.95,3.86) |
|  |  |  |  | #118 | 2.31(1.11,4.78) | #153 | 1.04(0.51,2.11) |
|  |  |  |  | #138 | 1.56(0.80,3.06) | #180 | 1.27(0.66,2.46) |
|  |  |  |  | #156 | 1.35(0.68,2.69) | #183 | 1.27(0.66,2.45) |
|  |  |  |  | #170 | 1.15(0.60,2.22) |  |  |
| Recio-Vega et al.,2011 | | Group IA^c^ | 1.19(0.81,1.70) | Group IIA | 1.22(0.99,1.49) | Group III | 1.81(1.08,3.04) |
|  |  | Group IB | 1.40(0.94,2.10) | Group IIB | 1.90(1.25,2.88) |  |  |
|  |  |  |  |  |  |  |  |

a: Group IB includes PCB congeners 177, 187 and 201; Group II includes 74,118,138, 156 and 170; Group III includes 153,180 and 183.

b: Group II includes PCB congeners 74,118,138,156,and170 ; Group III includes 153,180, and 183.

c: Group IA includes PCB congeners 44,52; Group IB includes congeners 101,187; Group IIA includes congeners 66,77,105,118 and 126; Group IIB

includes congeners 128,138 and170; Group III includes congeners 153 and 180.
