# Supplementary material for: Environmental Polychlorinated Biphenyl Exposure and Breast Cancer Risk: A Meta-Analysis of Observational Studies
Source: PLoS One. 2015 Nov 10;10(11):e0142513. doi: 10.1371/journal.pone.0142513 (PMC4640539; doi:10.1371/journal.pone.0142513)
Supplement: S3 Table — (DOC) [file pone.0142513.s009.doc]

**S3 Table. Summary of association between PCB exposure and breast cancer risk**

| PCBs | Number of studies | OR (95%CI) | *I^2^* (%) | P |
| --- | --- | --- | --- | --- |
|  |  |  |  |  |
| Total PCBs | 25 | 1.09(0.97,1.22) | 55.4 | 0.668 |
| Group I | 8 | 1.10(0.97,1.24) | 0.0 | 0.814 |
| Group II | 13 | 1.23(1.08,1.40) | 48.0 | 0.658 |
| Group III | 11 | 1.25(1.09,1.43) | 40.2 | 0.432 |

Abbreviations: OR, odds ratio; 95%CI, 95% conﬁdence interval; *I^2^*, index of heterogeneity; P, Egger test for publication bias
