# Supplementary material for: Environmental Polychlorinated Biphenyl Exposure and Breast Cancer Risk: A Meta-Analysis of Observational Studies
Source: PLoS One. 2015 Nov 10;10(11):e0142513. doi: 10.1371/journal.pone.0142513 (PMC4640539; doi:10.1371/journal.pone.0142513)
Supplement: S4 Table — (DOC) [file pone.0142513.s010.doc]

**S4 Table.** Evaluation of quality based on the Newcastle-Ottawa scale for the included studies.

**A.** Evaluation of quality based on the Newcastle-Ottawa scale for prospective epidemiologic (nested case-control) studies

|  | **Selection** | | | | **Comparability** | | **Outcome** | | | **Total** |
| --- | --- | --- | --- | --- | --- | --- | --- | --- | --- | --- |
| **Study** | Representativeness | Selection of non-exposed | Ascertainment of exposure | Outcome not present at start | On lipid | On other risk factors | Assessment of outcome | Long enough follow-up (median ≥5 years) | Adequacy (completeness) of follow-up |  |
| Laden et al., 2001 | 0 | 1 | 1 | 1 | 1 | 1 | 1 | 1 | 1 | 8 |
| Ward et al., 2000 | 0 | 1 | 1 | 1 | 1 | 0 | 1 | 1 | 1 | 7 |
| Wolff et al., 2000 | 1 | 1 | 1 | 1 | 1 | 1 | 1 | 1 | 1 | 9 |
| Helzlsouer et al., 1999 | 1 | 1 | 1 | 1 | 1 | 0 | 1 | 1 | 1 | 8 |
| Krieger et al., 1994 | 1 | 1 | 1 | 1 | 0 | 1 | 1 | 1 | 1 | 8 |
| Dorgan et al., 1999 | 0 | 1 | 1 | 1 | 1 | 1 | 1 | 1 | 1 | 8 |
| Cohn et al., 2012 | 0 | 1 | 1 | 1 | 0 | 1 | 1 | 1 | 1 | 7 |
| Høyer et al., 1998 | 1 | 1 | 1 | 1 | 1 | 1 | 1 | 1 | 0 | 8 |
| Raaschou-Nielsen et al., 2005 | 1 | 1 | 1 | 1 | 1 | 1 | 1 | 1 | 1 | 9 |

**B.** Evaluation of quality based on the Newcastle-Ottawa scale for retrospective epidemiologic (case-control) studies

|  | **Selection** | | | | **Comparability** | | **Exposure** | | | **Total** |
| --- | --- | --- | --- | --- | --- | --- | --- | --- | --- | --- |
| **Study** | Case definition | Representativeness of the cases | Selection of controls | Definition of controls | On lipid | On other risk factors | Assessment of exposure | Same method of ascertainment for cases and controls | Non-response rate |  |
| Zhang et al., 2013 | 1 | 1 | 0 | 1 | 0 | 1 | 0 | 1 | 1 | 6 |
| Itoh et al., 2009 | 1 | 1 | 0 | 1 | 1 | 1 | 0 | 1 | 1 | 7 |
| Gatto et al., 2007 | 1 | 1 | 1 | 1 | 1 | 1 | 0 | 1 | 0 | 7 |
| Rubin et al., 2006 | 1 | 0 | 1 | 1 | 1 | 1 | 0 | 1 | 1 | 7 |
| Charlier et al., 2004 | 1 | 0 | 1 | 1 | 0 | 1 | 0 | 1 | 1 | 6 |
| Lopez-Carrillo et al., 2002 | 1 | 0 | 0 | 1 | 1 | 1 | 0 | 1 | 1 | 6 |
| Zheng et al., 2000 | 1 | 1 | 1 | 1 | 1 | 1 | 0 | 1 | 0 | 7 |
| Moysich et al., 1998 | 1 | 1 | 1 | 1 | 1 | 1 | 0 | 1 | 1 | 8 |
| Gammon et al., 2002 | 1 | 1 | 1 | 1 | 1 | 1 | 0 | 1 | 1 | 8 |
| Wolff et al., 2000 | 1 | 0 | 0 | 1 | 1 | 1 | 0 | 1 | 1 | 6 |
| Ye et al., 2009 | 1 | 1 | 1 | 1 | 0 | 1 | 0 | 1 | 0 | 6 |
| Millikan et al., 2000 | 1 | 1 | 1 | 1 | 1 | 1 | 0 | 1 | 0 | 7 |
| Demers et al., 2002 | 1 | 0 | 0 | 1 | 1 | 1 | 0 | 1 | 1 | 6 |
| Stellman et al., 2000 | 1 | 0 | 0 | 1 | 1 | 1 | 0 | 1 | 1 | 6 |
| Aronson et al., 2000  Recio-Vega et al., 2011 | 1  1 | 0  0 | 0  0 | 1  1 | 1  0 | 1  1 | 1  0 | 1  1 | 0  1 | 6  5 |
