# Supplementary material for: Environmental Polychlorinated Biphenyl Exposure and Breast Cancer Risk: A Meta-Analysis of Observational Studies
Source: PLoS One. 2015 Nov 10;10(11):e0142513. doi: 10.1371/journal.pone.0142513 (PMC4640539; doi:10.1371/journal.pone.0142513)
Supplement: S5 Table — (DOC) [file pone.0142513.s011.doc]

**S5 Table. Characteristics of the excluded studies based on less than 50 breast cancer cases.**

| Study | Location | Study Period | No. of  cases: controls | Age  cases: controls | No. of measured congeners | Mean±SD of PCBs (ng/g lipid) | | OR (95%CI) | Adjustment for Covariates | Biologic  specimen |
| --- | --- | --- | --- | --- | --- | --- | --- | --- | --- | --- |
|  |  |  |  |  |  | Cases | Controls |  |  |  |
| Bonefeld-Jorgensen, E. C.  et al., 2011 | Greenlandic Inuit | 2000-2003 | 31:115  (P) | 50^a^ | 12 | 2049^b^ | 1867^b^ | Total PCBs 1.00(1.00; 1.00) | not adjusted since no confounder was identified. | serum |
| Pavuk, M.  et al.,2003 | Eastern Slovakia | 1997-1999 | 24:88  (P) | 51.5:46.0^a^ | 15 | 2586 ^b^ | 2682^b^ | Total PCBs 0.42(0.10–1.82)  Group I ^b^  0.37(0.10–1.43)  Group II ^c^  0.32(0.07–1.56)  Group III ^d^  0.49(0.12–2.04) | age, age at menarche, education (r8, 9–10, >10 years), alcohol consumption (0, 1–3, >3 drinks a week), and pack-years of smoking. | serum |
| Liljegren, G. et al., 1998 | Sweden | 1993-1995 | 43:35  (H) | 57.7:53.7^a^ | 3 | 1205 | 1149 | Total PCBs 0.7(0.1-2.4) | age, parity, heredity, smoking. | adipose tissue |

Abbreviations: OR, odds ratio; CI ,confidence intervals; SD, standard deviation; H, hospital control group; P, population control group.

a: median

b: PCB congeners 28, 52, 101.

c: PCB congeners 105, 114, 118, 123, 156, 157, 167, 189.
d: PCB congeners 138, 153, 170, 180.
